# Decellularized ovarian bioscaffolds and resveratrol-loaded polymeric nanoparticles support in vitro viability and ultrastructural preservation of bovine preantral follicles

**DOI:** 10.1007/s10815-026-03823-3

**Published:** 2026-03-17

**Authors:** Francisco das Chagas Costa, Ernando Igo Teixeira de Assis, Danisvânia Ripardo Nascimento, Miguel Fernandes de Lima Neto, Andreza Aguiar Silva, Venância Antônia Nunes Azevedo, Regislane Pinto Ribeiro, Bianca Régia Silva, Mariana Aragão Matos Donato, Alice Vitória Frota Reis, Josimar Oliveira Eloy, José Roberto Viana Silva

**Affiliations:** 1 Laboratory of Biotechnology and Reproductive Physiology, Federal University of Ceará, Sobral, Brazil; 2 State University of Ceará - UECE, Fortaleza, Brazil; 3 Federal University of Pernambuco, Recife, Brazil; 4 Department of Pharmacy, Faculty of Pharmacy, Dentistry and Nursing, Universidade Federal do Ceará, Fortaleza, Brazil

**Keywords:** Decellularization, Ovarian follicles, Nanotechnology, Resveratrol, Antioxidant

## Abstract

**Purpose:**

This study evaluated the effects of decellularized ovarian bioscaffolds and resveratrol-loaded polymeric nanoparticles (RLPN) on the in vitro culture, viability, and ultrastructural preservation of bovine secondary follicles.

**Methods:**

Decellularized extracellular matrix (dECM) from cortical fragments was obtained by freeze–thaw cycles and sequential incubation in Triton X-100 and sodium dodecyl sulfate. Decellularization efficiency and extracellular matrix integrity were assessed by hematoxylin–eosin, Hoechst staining, scanning electron microscopy, and quantification of collagen and glycosaminoglycans. Bovine secondary follicles were isolated and cultured for 12 days in either a two-dimensional (2D) system or in dECM scaffolds in medium supplemented with 0.02, 0.2, or 2.0 µM RLNP, blank nanoparticles, or unencapsulated resveratrol. Follicular viability and ultrastructure were evaluated by calcein-AM/ethidium homodimer-1 staining and transmission electron microscopy. Expression of mRNA for catalase, superoxide dismutase, glutathione peroxidase 1, peroxiredoxin 6, and nuclear factor erythroid 2-related factor 2 was assessed by qRT-PCR. Quantitative data were analyzed by unpaired t-tests or one-way ANOVA, followed by Tukey’s test (*P* < 0.05).

**Results:**

Hematoxylin–eosin and Hoechst staining confirmed effective cell removal, while collagen, glycosaminoglycans, and ECM ultrastructure were preserved. Follicles cultured in the three-dimensional (3D) system showed increased viability, further enhanced by 0.02 or 2.00 µM RLPN. Follicles cultured with 0.02 µM RLPN exhibited well-preserved morphology, including intact zona pellucida, oocyte membrane, and organelles. qRT-PCR analysis revealed reduced mRNA expression of antioxidant-related genes in follicles cultured with RLNP.

**Conclusion:**

The decellularization protocol effectively removed cellular content and preserved ECM structure and ultrastructure. 3D culture system combined with supplementation of 0.02 µM RLNP supported follicular viability and ultrastructural preservation and was associated with transcriptional changes in antioxidant-related genes.

## Introduction

Most of the ovarian follicles are lost due to atresia, which is significantly increased beyond the secondary follicular stage in bovine species [1, 2]. This physiological follicular depletion reinforces the need for improvement of reproductive biotechnologies capable of utilizing the pool of early follicles. The in vitro culture (IVC) of secondary follicles isolated from bovine ovaries is a key tool to elucidate their specific requirements for development up to maturation stages and to improve the use of ovarian reserve by assisted reproductive technologies [3]. Despite efforts to establish a suitable IVC system for secondary follicles [4, 5], obtaining matured oocytes from isolated bovine secondary follicles cultured in vitro remains inefficient as only a limited proportion of follicles progress beyond early growth stages, and morphological development is often not accompanied by the coordinated acquisition of functional oocyte competence [6–8]. These limitations may reduce the effective use of the ovarian reserve and hamper the translational application of in vitro follicle culture systems for reproductive biotechnologies and fertility preservation, underscoring the challenge of reproducing the physiological conditions required for complete follicular development.

Recent evidences have shown that follicles are highly responsive to biomechanical signals originating from the ovarian stroma that act during folliculogenesis (Wang et al., [9]). The coordinated activity of these mechanical cues, along with endocrine and paracrine signaling, shapes individual cells, oocytes, and the entire follicle [10]. Thus, after isolation, secondary follicles are challenging to in vitro culture without the supporting structure of the ovary [11]. Therefore, biocompatible scaffolds to encapsulate isolated follicles can provide a suitable environment for their in vitro development [12]. Ovarian follicles have been cultured in a three-dimensional culture system using different biomaterials, such as alginate [13], collagen [14], fibrin [15], and polyethylene glycol [16]. However, none of these materials have demonstrated the ability to fully replicate all the functions of the native ovarian ECM, including cellular support and regulatory roles [17]. Several studies have proposed the use of decellularized ovarian extracellular matrix (dECM) as a natural scaffold that closely mimics the native ovarian extracellular matrix (ECM) [18]. The ovarian cortex represents the native microenvironment of preantral follicles and provides specific biomechanical and biochemical cues essential for their maintenance and early development [19]. For instance, Laronda et al. [20] showed that bovine ovarian dECM positively influenced murine primary ovarian cells and stimulated the formation of follicle-like structures in vitro. However, the effect of dECM on the in vitro development of isolated follicles in bovine species remains unexplored.

In vitro redox dysregulation is also correlated with poor follicular quality after culturing [6, 21, 22]. During in vitro culture of follicles, various factors, such as ischemia, ovarian fragmentation, manipulation, and light exposure can increase the generation of reactive oxygen species (ROS) that leads to oxidative stress [23]. Elevated ROS levels are linked with a reduction in follicular viability, lipid peroxidation leading to changes in membrane permeability and selectivity (Gaschler and Stockwell, [24]), vacuolization and degeneration of mitochondrial cristae and matrix [25], endoplasmic reticulum stress [26], and zona pellucida (ZP) damage [27]. Consequently, antioxidant supplementation of the culture medium has been proposed to maintain the redox balance during in vitro ovarian follicles culture [28]. Moreover, the expression levels of enzymatic antioxidants such as catalase (*CAT*), superoxide dismutase (*SOD*), glutathione peroxidase (*GPX*), peroxiredoxin (*PRDX*), and nuclear factor erythroid 2-related factor 2 (*NRF2*) are frequently used as indicators of the antioxidant capacity of in vitro culture systems [6, 29–32].

Among the natural phenolic compounds, resveratrol is known for improving follicular development in vitro through its anti-apoptotic and antioxidant properties [23, 33, 34]. Although promising results have been reported, its low water solubility (~ 3 mg/100 mL) and rapid metabolism can impact its bioavailability, limiting the extent to which its free form exerts its effects [35, 64]. Nanotechnology-based delivery systems have been proposed as a strategy to enhance resveratrol stability and bioavailability, potentially allowing biological effects to be achieved at lower concentrations during in vitro culture. In this context, encapsulating resveratrol within polymeric nanoparticles is a promising strategy, as it can enhance the effectiveness of its action. The advantages of polymeric nanoparticles include superior biocompatibility compared to other materials, minimal toxicity, capable of sustained drug release, and biodegradability [64].

This study aimed to decellularize and characterize bovine ovarian tissue and to evaluate its effect on the follicle viability and ultrastructure, and expression of mRNA for *CAT, SOD, GPX1, PRDX6*, and *NRF2* in bovine secondary follicles cultured in medium supplemented with different concentration of in vitro resveratrol-loaded polymeric nanoparticles.

## Material and methods

### Chemicals

Culture media and other chemicals used in the present study were purchased from Sigma-Aldrich (St. Louis, MO, USA), unless otherwise specified in the text.

### Synthesis and physicochemical characterization of resveratrol-loaded polymeric nanoparticles

Resveratrol-loaded polymeric nanoparticles (RLNP) corresponding to the NP04 formulation described by [64] were prepared by the nanoprecipitation method. Briefly, resveratrol and poly-ε-caprolactone (PCL; Mw ≈ 14,000) were dissolved in acetone to form the organic phase, which was slowly added dropwise into an aqueous phase composed of phosphate-buffered saline (PBS, pH 7.4) containing 0.15% (w/v) Poloxamer 407, under constant stirring. The organic solvent was subsequently evaporated, yielding stable polymeric nanoparticles. The mean particle size, polydispersity index (PDI), and zeta potential were evaluated to characterize the physicochemical properties of the formulations. For this purpose, the hydrodynamic size distribution, polydispersity index (PDI), and zeta potential of the nanoparticles were analyzed at 25 °C by dynamic light scattering (DLS). Measurements were conducted with a Malvern Zetasizer Nano ZS (Malvern Instruments, Malvern, UK), equipped with a 4 mW He–Ne laser at a wavelength of 633 nm at an angle of 90°. Prior to analysis, samples were diluted 1:10 in ultrapure water and vortexed to ensure proper dispersion. All measurements were performed in triplicate, and results were expressed as mean ± standard deviation (SD). The encapsulation efficiency, in vitro release profile, and nanoparticle morphology assessed by scanning electron microscopy were previously evaluated and reported by [64].

### Bovine ovaries and ethical approval

Ovaries from 50 healthy mixed-breed cows of reproductive age were collected at local slaughterhouses and assigned to two distinct experiments, as detailed below. All procedures performed in this study were approved by the Ethics and Animal Welfare Committee of the Federal University of Ceará under approval number 04/22. Immediately after collection, the ovaries were washed once with 70% alcohol and twice with PBS buffered with 20 mM HEPES and supplemented with 100 µg/ml penicillin and 100 µg/ml streptomycin, then designated for decellularization. The ovaries to be used for follicular isolation were washed twice in TCM199 buffered with 20 mM HEPES and supplemented with the same antibiotics. Then, they were transported to the laboratory within 1 h in the respective washing solution at 4 °C.

### Bovine ovarian tissue decellularization

Upon arrival at the laboratory, the ovaries (n = 10 pairs) were rinsed in PBS solution and stored at − 80 °C for 24–48 h. Before use, they were thawed in water bath at 37 °C for 1 h to ensure complete and uniform thawing of the bovine tissue prior to sectioning and decellularization, as previously described for Pennarossa et al. [36]. After thawing, antral follicles were punctured with a needle, and cortical slices (2 mm × 2 mm × 1 mm) were taken using a scalpel. Random samples from each ovarian pair were fixed in 4% paraformaldehyde prepared in PBS (pH 7.4) for 24 h at room temperature for evaluation as native tissue. Two decellularization solutions (DS) were prepared 24 h prior to use: (DS1)—1% Triton-X100 (v/v) (T8787-100 ML, Sigma-Aldrich, USA) in distilled water,(DS2)—0.5% SDS (w/v) (L3771-100 G, Sigma-Aldrich, USA). Initially, samples were immersed in 20 mL DS1 for 9 h under constant agitation (150 rpm) in an orbital shaker at 37 °C, followed by 9 h in 20 mL DS2 under the same conditions. After decellularization, the samples were washed 10 times in 50 mL PBS at 37 °C with hourly solution changes. The resulting samples were either used to assess the decellularization efficiency or stored at − 80 °C.

### Histological analysis and DNA staining

Native bovine ovarian tissue and decellularized extracellular matrix (dECM) morphology were analyzed using classical light microscopy. Samples were fixed for 24 h at room temperature and processed for histological. Sections (6 μm thick) were mounted on glass slides and stained with hematoxylin–eosin (H&E) to assess the remaining cell content after decellularization. Some sections were stained with 20 μM Hoechst 33,342 (B2261-100MG, Sigma-Aldrich, USA) for 1 min for DNA fluorescence labeling. For cell counting, random fields from sections were analyzed at × 400 magnification using a Nikon Eclipse TS 100 microscope and the ImageJ software (Version 1.54f, 2023). Cells were manually counted in a 100 μm^2^ area, following Silva et al. (2024a, b), with all measurements performed by a single operator. Images of sections stained with Hoechst 33,342 under constant exposure settings and analyzed with ImageJ. Residual nuclear content was considered proportional to the fluorescence intensity after subtraction of tissue auto fluorescence [11].

### Histochemical evaluation for collagen and glycosaminoglycans

For collagen assessment, 6 μm-thick sections from both native and decellularized bovine ovarian samples were dewaxed using xylene and stained with 0.1% Sirius Red solution for 1 h at room temperature, according to the protocol described by Rittié et al. ([37]). The excess stain was eliminated with a 0.5% acetic acid solution, followed by dehydration of the sections and subsequent mounting on slides. For glycosaminoglycan (GAG) evaluation, the sections were stained with Alcian Blue for 5 min at room temperature, following the manufacturer’s protocol. After staining, the sections were dehydrated and mounted on slides for analysis. For both collagen and GAG assessments, images from 50 fields (5 per animal) were captured using a DS Cooled DS-Ri1 camera attached to a Nikon Eclipse TS 100 microscope (Tokyo, Japan) at 400 × magnification. Quantification of collagen and GAG content was performed using Fiji-ImageJ software (Version 1.54f, 2023).

### Scanning electron microscopy (SEM)

For SEM, both native bovine ovaries and dECM samples were fixed in Karnovsky’s solution at 4 °C for 24 h, followed by post-fixation with 2% osmium tetroxide for 1 h. After washing with distilled water, the samples underwent gradual dehydration through a graded acetone series (30%, 50%, 70%, 90%, and 100%), with each step lasting 15 min. Subsequently, the specimens were dried using a critical point dryer, mounted on stubs, coated with colloidal gold, and observed using a scanning electron microscope (Inspect S50-FEI).

### Follicle isolation and in vitro culture

A total of 40 bovine ovaries were collected following the previously described procedures. The ovarian cortex was sectioned into 1–2 mm fragments using a sterile scalpel blade and transferred to TCM-199 medium supplemented with 100 µg/ml penicillin, 100 µg/ml streptomycin, and HEPES buffer. Secondary follicles measuring approximately 150–200 µm in diameter were manually dissected from the cortical strips using 26-gauge needles under a stereomicroscope (SMZ 645 Nikon, Tokyo, Japan). Follicles selected for culture exhibited an intact basement membrane, two or more granulosa cell layers, absence of an antral cavity, and a morphologically healthy, round oocyte centrally located within the follicle.

To culture the follicles, the decellularized scaffolds were removed from the − 80 °C freezer and thawed for 1 h in a water bath containing distilled water. Subsequently, the structures were sterilized in a PBS solution with penicillin and streptomycin, then placed in TCM-199 medium supplemented with penicillin (100 µg/ml), streptomycin (100 µg/ml) and HEPES solution, and stored in an incubator at 38.5 °C with 5% CO2 for 4 h prior to use. The base medium used for follicle culture was TCM-199 (pH 7.2–7.4), enriched with 3.0 mg/ml bovine serum albumin (BSA), 2 mM glutamine, 2 mM hypoxanthine, 100 IU/ml penicillin–streptomycin, 10 µg/ml insulin, 5.5 µg/ml transferrin, 5 ng/ml selenium (ITS), 50 µg/ml ascorbic acid, and 100 ng/ml equine chorionic gonadotropin (eCG) (TCM-199^+^). Isolated secondary follicles were randomly distributed and cultured in two systems: (I) two-dimensional (2D) system, i.e., the follicles were cultured in Petri dishes (60 × 15 mm; Corning, USA) in drops of 100 µL TCM-199^+^ (TCM199^+^2D); (II) three-dimensional (3D) system, i.e., the follicles were inserted in dECM scaffolds and cultured in 24-well culture dishes (2 scaffolds per well) in 500 µL TCM-199^+^ alone (TCM199^+^3D) or supplemented with 0.02, 0.2, or 2.0 µM resveratrol-loaded polymeric nanoparticles (RLNP-3D groups), 2.0 µM blank nanoparticles BLNP (2 µM-3D); or 2.0 µM non-encapsulated resveratrol RSV (Free-RSV-3D). The concentrations of resveratrol-loaded polymeric nanoparticles were defined based on previous studies using free resveratrol in in vitro follicle culture [34]. Follicles were cultured for 12 days in a humidified incubator at 38.5 °C with 5% CO₂ in air. Half of the culture medium was refreshed every second day of culture.

### Assessment of follicular viability by fluorescence microscopy

Following the culture period, follicles (n = 30 per treatment) were manually retrieved from the dECM scaffolds using 26-gauge needles. They were then incubated for 15 min at 37 °C with 5% CO₂ in 100 μL droplets of TCM-199 medium containing 4 μM calcein-AM and 2 μM ethidium homodimer-1 (EthD-1) (Molecular Probes—L3224, Invitrogen, Karlsruhe, Germany) to assess esterase activity in the cytoplasm and the labeling of nucleic acids in non-viable cells following membrane disruption. After staining, follicles were washed three times in TCM-199 medium and analyzed under a fluorescence microscope (Nikon, Eclipse TS100, Japan) as described by Paulino et al. [8]. Oocytes and granulosa cells with preserved viability displayed green fluorescence from calcein-AM, whereas non-viable cells exhibited red fluorescence due to EthD-1 staining. Fluorescence intensity was quantified using ImageJ software (Version 1.54f, 2023), considering the follicle as the experimental unit and preserving follicular structural integrity. The mean pixel intensity within the follicular region was measured after background correction to determine staining levels. Non-cultured follicles were used as the reference for relative fluorescence quantification, following the approach described by Rocha-Frigoni et al. [38].

### Morphological and ultrastructural assessments of dECM, and cultured follicles

To assess the structural stability of collagen and glycosaminoglycan (GAG) networks throughout the culture period, dECM scaffolds were stained with Picrosirius red and Alcian blue, respectively, on days 0, 2, 4, 6, 8, 10, 12, and 14. Additionally, scaffold ultrastructure was examined by scanning electron microscopy (SEM) on day 12 of culture. Histochemical and SEM processing were performed using standard methods already described previously. To analyze cell morphology and organelle organization in oocytes and GCs, transmission electron microscopy (TEM) was performed on follicles cultured in the 2D system or in the 3D system in medium supplemented with 0.02, 0.2, or 2.0 µM RLNP, Blank-NP, or Free-RSV. After culture, scaffolds containing follicles (6–10 per treatment) were fixed in Karnovsky’s solution for 4 h at room temperature (~ 25 °C), embedded in 4% low-melting agarose droplets, and kept in sodium cacodylate buffer. As no significant differences in viability were found among different concentrations of RLNP, follicles treated with the lowest concentration (0.02 µM) were selected for ultrastructural analysis. The specimens were post-fixed with 1% osmium tetroxide, 0.8% potassium ferricyanide, and 5 mM calcium chloride. After dehydration in acetone, samples were embedded in epoxy resin (Epoxy Embedding Kit, Fluka Chemika). Semithin Sects. (2 μm) were stained with toluidine blue and examined under light microscopy at 400 × magnification, while ultrathin Sects. (70 nm) were counterstained with uranyl acetate and lead citrate for examination under a Morgani-FEI transmission electron microscope.

#### Quantitative reverse transcription-polymerase chain reaction (qRT-PCR)

Real-time PCR was used to evaluate the levels of mRNAs for *CAT, SOD, GPX1*, *PRDX6*, and *NRF2* in follicles cultured follicles 2D system or in 3D system in medium supplemented with 0.02 µM RLNP, Blank-NP or Free-RSV, according to Azevedo et al. [6]. After culture, follicles were carefully removed from dECM scaffolds, and total mRNA was extracted using the Trizol purification kit (Invitrogen, São Paulo, Brazil), according to the manufacturer’s guidelines. The total mRNA concentration was measured using a nanodrop (Biodrop, Cambridge, England), and 50 ng/µL of mRNA was used for reverse transcription. Quantification of mRNA was conducted using SYBR Green on a StepOnePlus instrument (Applied Biosystems, Foster City, CA, USA). Each quantitative PCR reaction (15 µL total volume) was prepared with 7.5 µL of SYBR Green Master Mix (PE Applied Biosystems, Foster City, CA), 5.5 µL of ultrapure water, 1 µL of cDNA, and 0.5 µM of each primer (Table 1). The relative expression levels of all target genes were normalized to the endogenous control gene Glyceraldehyde-3-phosphate dehydrogenase (GAPDH) [39] calculated using the 2^-ΔΔCt method, as described by Cao et al. [40].

**Table 1:** Oligonucleotide primers used for polymerase chain reaction analysis

Target gene	Primer sequence (5′ ➔ 3′)	Sense (S), anti-sense (As)	GenBank accession no
*GAPDH*	TGTTTGTGATGGGCGTGAACCA ATGGCGCGTGGACAGTGGTCATAA	S As	GI: 402744670
*CAT*	AAGTTCTGCATCGCCACTCA GGGGCCCTACTGTCAGACTA	S As	GI: 402693375
*SOD*	GTGAACAACCTCAACGTCGC GGGTTCTCCACCACCGTTAG	S As	GI: 31341527
*GPX1*	AACGTAGCATCGCTCTGAGG GATGCCCAAACTGGTTGCAG	S As	GI: 156602645
*PRDX6*	GCACCTCCTCTTACTTCCCG GATGCGGCCGATGGTAGTAT	S As	GI: 59858298
*NRF2*	GACCCAGTCCAACCTTTGTC GACCCGGACTTACAGGTACT	S As	GI: 0304941

### Statistical analysis

Statistical analyses were performed using GraphPad Prism version 9.0. An unpaired t-test was applied to compare cell remnants and the percentage of collagen and GAGs between native and decellularized tissues. For treatment group comparisons, data with normal distribution were evaluated using one-way ANOVA followed by Tukey’s post hoc test, while non-normally distributed variables were assessed with the Kruskal–Wallis test and Dunn’s multiple comparisons. Results are presented as mean ± standard error of the mean (SEM), unless otherwise specified. Differences were considered statistically significant when *P* < 0.05.

## Results

### Physicochemical characterization confirms successful synthesis of RLNP

Table 2 shows the physicochemical characterization of resveratrol-loaded polymeric nanoparticles (RLNP) and blank nanoparticles (BLNP). The synthesis method employed is robust and effective in producing nanoparticles with sizes below 150 nm. RLNP showed a PDI of 0.26, while BLNP presented a PDI of 0.16, indicating moderate size distribution uniformity. Both formulations exhibited a negative surface charge, with zeta potential values close to − 6 mV, reflecting low surface charge intensity.

**Table 2:** Physicochemical properties of the formulations, including particle size, polydispersity index (PDI), and zeta potential

	Size (nm) ± SD	PDI ± SD	Zeta potential (mV) ± SD
RLNP	130.87 ± 36.36	0.26 ± 0.01	−5.80 ± 1.26
BLNP	142.23 ± 1.55	0.16 ± 0.01	−5.65 ± 1.85

### Decellularization eliminates cells and maintains tissue macro- and microarchitecture

**Fig. 1:**
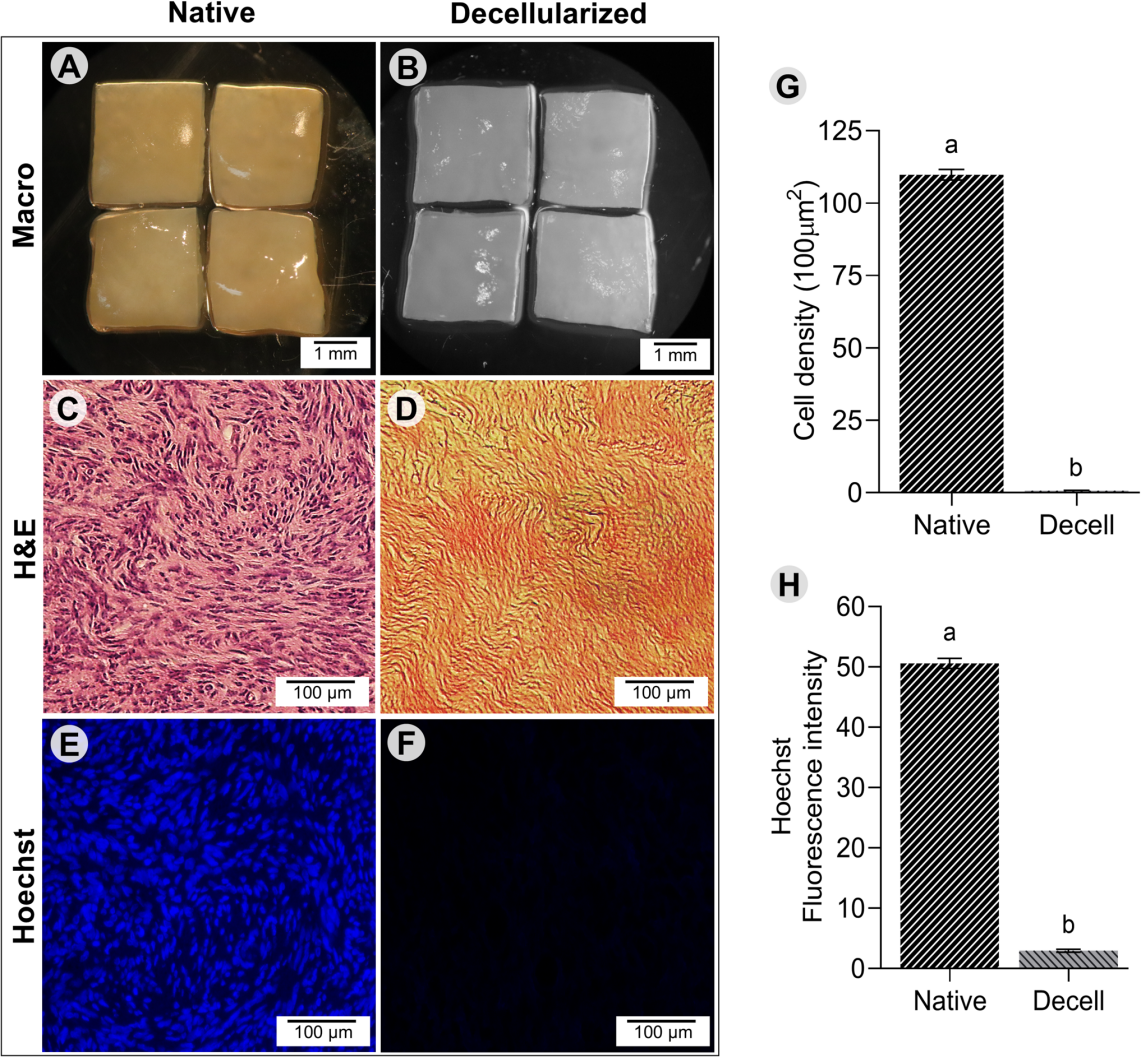
Macroscopic images of bovine ovarian tissue from the native control group (**A**) and decellularized extracellular matrix (dECM) group (**B**) (magnification = 25 ×; scale bar = 1 mm). Representative hematoxylin–eosin staining of native (**C**) and dECM (**D**) ovarian tissues, and Hoechst staining of native (**E**) and dECM (**F**) tissues (magnification = × 400; scale bar = 100 μm). Quantification of stromal cell density (**G**) and Hoechst fluorescence intensity (**H**) in native and dECM ovarian tissues. a, b, c: Different lowercase letters indicate statistically significant differences between treatments (*P* < 0.05)

**Fig. 2:**
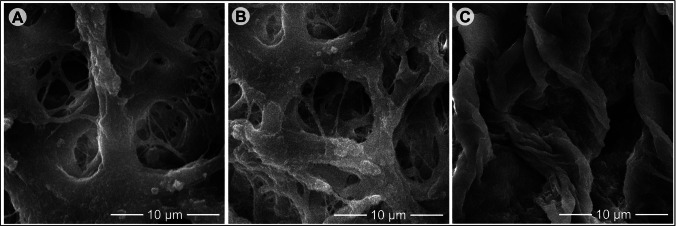
Scanning electron micrographs showing the microarchitecture of native ovarian tissue (**A**), dECM immediately after decellularization (**B**), and after 12 days in culture (**C**). Magnification = × 5000; scale bar = 10 μm

Macroscopic analysis showed that decellularized tissues changed color from red to white and maintained their shape and homogeneity without any signs of deformation (Fig. 1A, B). The SEM revealed that ECM fiber integrity was preserved after decellularization. The fiber appearance and organization in the dECM closely resembled those of the native ovarian tissue. The SEM also confirmed the preservation of both dense and thin collagen fibers, indicating that the resulting scaffolds retained their native three-dimensional architecture (Fig. 2A–C). Histological analysis revealed that the resulting ECM-based scaffolds were devoid of cells and basophilic staining was absent in the decellularized scaffolds, whereas cell nuclei were distinctly visible in native tissues, which served as the control (Fig. 1C, D). Cell density analysis demonstrated absence of nuclei in the ECM-based scaffolds compared to the untreated tissues (*P* < 0.05) (Fig. 1G). Additionally, the existence of DNA was verified through Hoechst 33,342 staining (Fig. 1E, F) indicating absence of DNA in decellularized tissues (*P* < 0.05) (Fig. 1H).

### Decellularization preserves ovarian extracellular matrix components

**Fig. 3:**
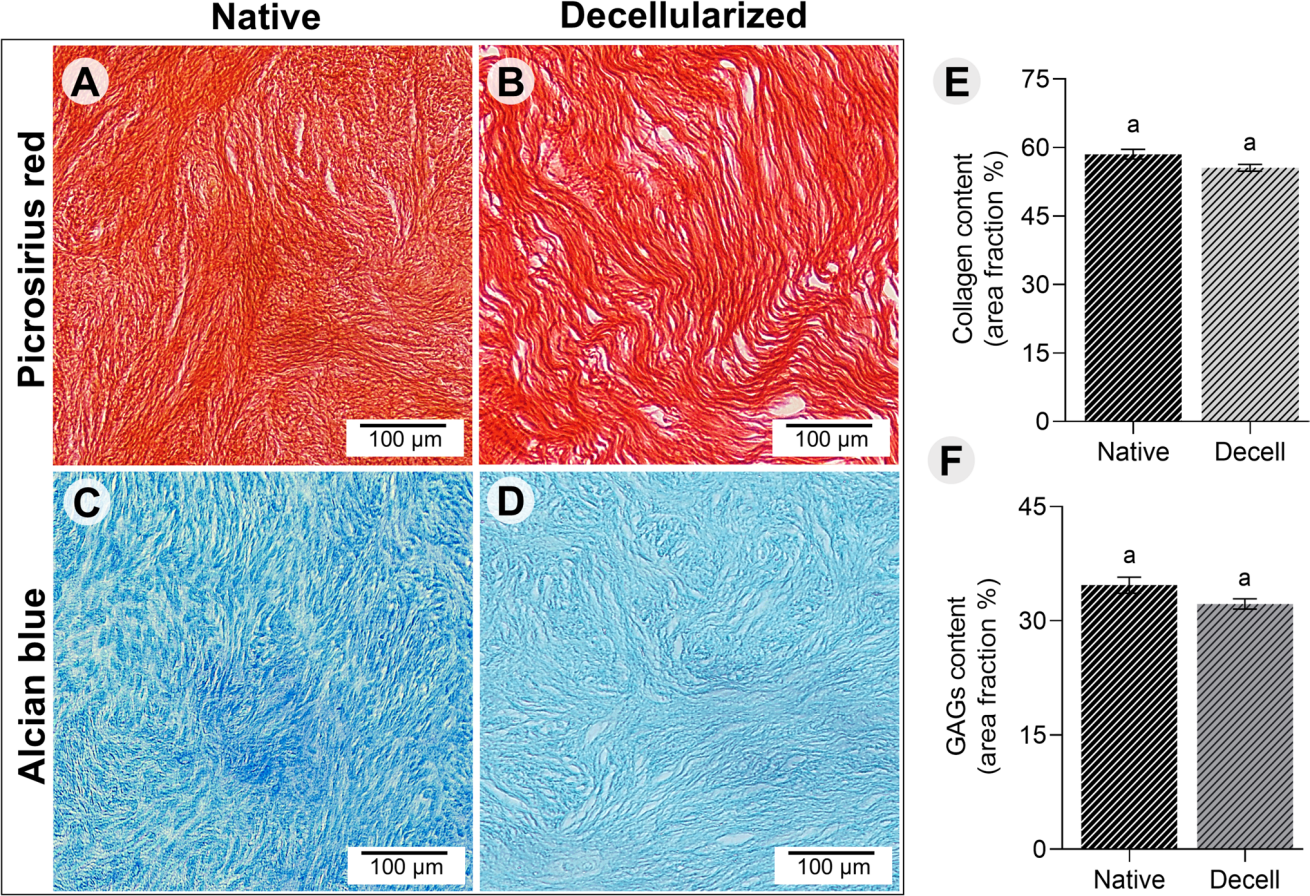
Histochemical staining of native (control group) and decellularized bovine ovarian tissue (dECM group). Picrosirius red staining for collagen (**A**, **B**), and Alcian blue staining for glycosaminoglycans (**C**, **D**) (magnification: × 400; scale bar = 100 μm). Quantification of collagen content (**E**), and glycosaminoglycans (**F**). a, b, c Different lowercase letters indicate statistically significant differences between treatments (*P* < 0.05)

Histochemical analyses confirmed the preservation of key ECM components after tissue decellularization. Picrosirius red and Alcian blue staining revealed the persistence of collagen (Fig. 3A, B) and GAGs (Fig. 3C, D), displaying a comparable distribution between ECM-based scaffolds and native tissues. These morphological findings were confirmed by stereological quantifications, which demonstrated no differences in collagen (Fig. 3E), and GAGs content (Fig. 3F) between ECM-based scaffold and native tissues (*P* < 0.05). The mean of collagen and GAGs in the decellularized ovarian tissue was about 95% and 93%, respectively, compared with native tissue collagen and GAGs content prior to decellularization.

### The dECM undergoes time-dependent structural changes in vitro

**Fig. 4:**
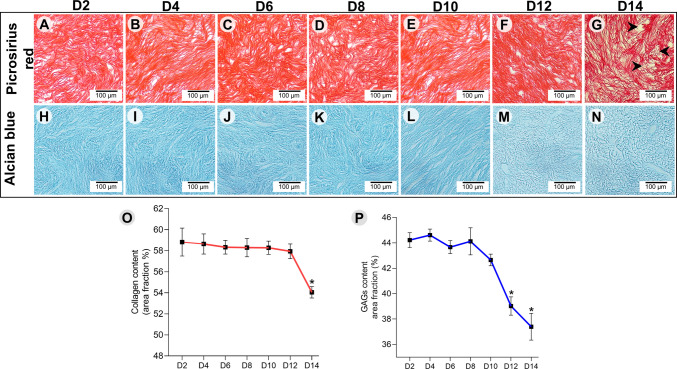
Structural remodeling of collagen **(A–G)** and GAGs **(H–N)** in decellularized ovarian scaffolds over 14 days of in vitro culture. Quantitative analysis of collagen **(O)** and GAG content **(P)** throughout the culture period. Black arrowheads indicate collagen fiber disruption points on day 14. Magnification = × 400; scale bar = 100 μm. Asterisks indicate statistically significant differences between time points (*P* < 0.05)

Picrosirius red staining showed that the total collagen content remained stable until day 12 of in vitro culture, with no apparent disarray in the collagen fiber bundles of the decellularized scaffolds. However, by day 14, a greater degree of fiber disorganization became evident, characterized by the presence of gaps, increased fiber fragmentation, and a significant reduction in fiber density within the scaffolds (Fig. 4A–G and O). Similarly, GAGs stained with Alcian blue remained quantitatively stable until day 10 of culture, although the GAG network appeared straighter at this stage. From day 12 onward, a significant decline in GAG density was observed, accompanied by a more dispersed network with an increased presence of gaps (Fig. 4H–N, and P). The SEM analysis corroborated the histochemical findings, revealing alterations in the ultrastructural integrity of the dECM after 12 days in culture. The finer fibers present in the native tissue and immediately after decellularization were no longer observed. Additionally, the porous architecture present shortly after decellularization was no longer apparent after 12 days of culture (Fig. 2C).

### The dECM and RLNP enhance follicular viability

**Fig. 5:**
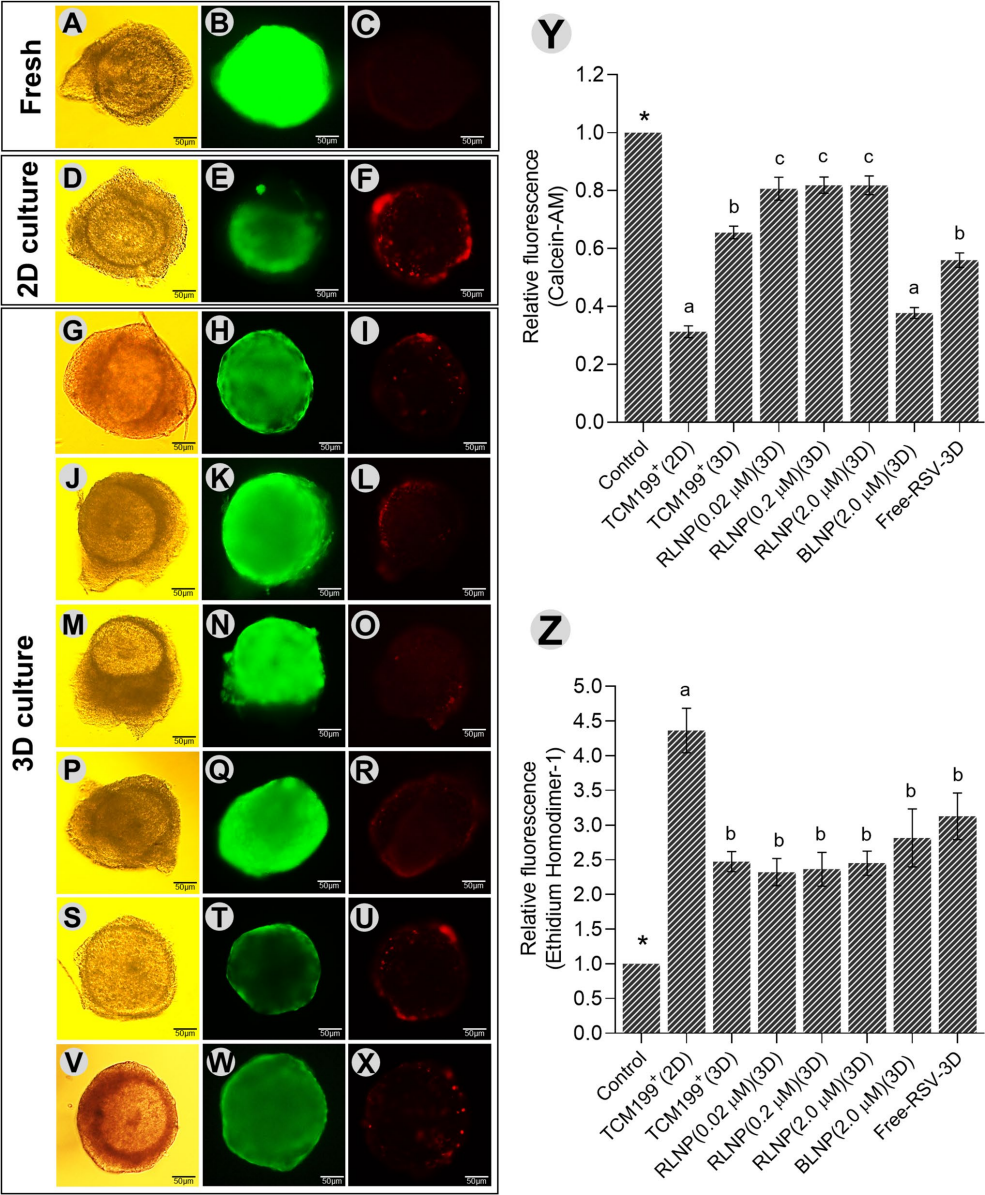
Representative images of secondary follicles in bright field (left column), stained with calcein-AM (middle column), or EthD-1 (right column): fresh control (**A**–**C**); 2D system with TCM-199 alone (**D**–**F**); 3D system with TCM-199 alone (**G**–**I**); or supplemented with 0.02 (**J**–**L**), 0.2 (**M**–**O**), or 2 µM (**P**–**R**) resveratrol-loaded nanoparticles, blank nanoparticles (**S**–**U**), or unencapsulated resveratrol (**V**–**X**). Quantification of calcein-AM (**Y**) and EthD-1 (**Z**) fluorescence. Scale bar = 50 µm

After culture, follicles in all treatments and culture conditions exhibited a significant reduction in calcein-AM fluorescence intensity and an increase in EthD-1 fluorescence compared to the fresh control (Fig. 5A–X**)**. Follicles cultured in the 3D groups, except those supplemented with BLNP, exhibited higher calcein-AM fluorescence intensity compared to the 2D group. Meanwhile, lower EthD-1 fluorescence intensity was observed in all the 3D groups relative to the 2D group (*P* < 0.05), (Fig. 5Y, Z). In follicles cultured in 3D system, supplementation of the culture medium with 0.02, 0.2, and 2.0 µM RLNP further increased calcein-AM fluorescence intensity compared to the other treatments, with no significant differences observed among the three concentrations (Fig. 5Y). However, this supplementation did not significantly affect EthD-1 fluorescence intensity (*P* > 0.05) (Fig. 5Z).

### The dECM and RLNP improve follicular ultrastructure

**Fig. 6:**
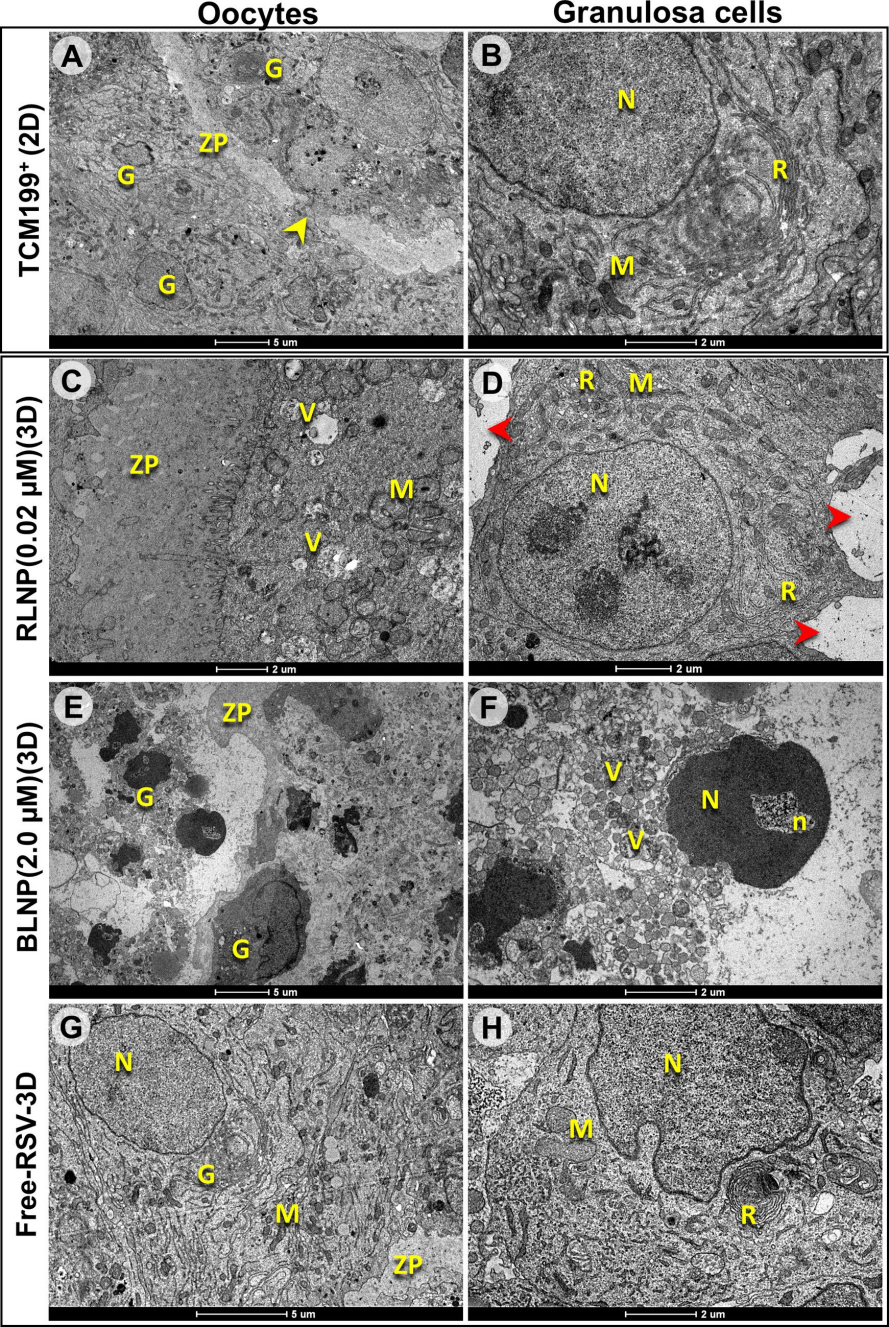
Electron micrograph of oocyte (left column), and granulosa cells (right column) from secondary follicles cultured in 2D system in TCM-199^+^ (**A**, **B**) or in 3D system in TCM-199^+^ supplemented with resveratrol-loaded nanoparticles 0.02 μM (**C**, **D**), blank nanoparticles (**E**, **F**) or nonencapsulated resveratrol (**G**, **H**). Red arrowheads indicate gaps between adjacent granulosa cells. Scale bar = 5 μm (**A**, **E**, and **G**); 2 μm (**B**, **C**, **D**, **F**, and **H**). Symbols: ZP, zona pellucida; G, granulosa cells; N, nucleus; M, mitochondria; R, reticulum; V, vacuoles; n, nucleolus

Follicles cultured in the 2D system exhibited a ruptured ZP, with the space originally occupied by the oocyte filled by granulosa cells. However, these cells were well preserved, displaying reticulum and mitochondria, although mitochondrial cristae were difficult to visualize (Fig. 6A, B). In contrast, follicles cultured in the 3D system in medium supplemented with 0.02 μM RLNP exhibited oocytes with an intact ZP and visible microvilli. Moreover, mitochondria with preserved cristae were observed. Although some granulosa cells showed gaps between them, they remained well preserved, with numerous mitochondria featuring clearly visible cristae, along with the presence of endoplasmic reticulum (Fig. 6C, D). In follicles cultured in the 3D system in medium supplemented with 2.0 μM BLNP, although remnants of an irregular ZP were visible, no viable oocyte was identified. Granulosa cells of these follicles displayed intense vacuolization, an extremely heterochromatic nucleus, and heterogeneous nucleoli. Furthermore, an intact plasma membrane separating the cells was not observed (Fig. 6E, F). Finally, in follicles cultured in the 3D system with 2.0 μM Free-RSV, no intact oocyte was identified, with only fragments of the ZP remaining. However, granulosa cells had preserved morphology, with a well-defined nucleus, mitochondria with reasonably preserved cristae, and structurally intact endoplasmic reticulum (Fig. 6G–H).

### The RLNP downregulated mRNA expression of antioxidant enzymes

**Fig. 7:**
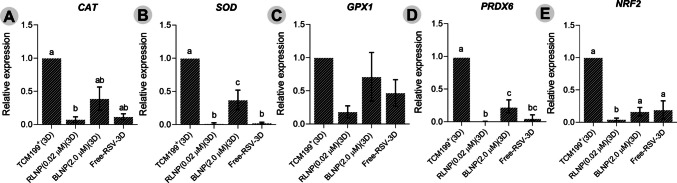
Relative mRNA expression levels of *CAT* (**A**), *SOD* (**B**), *GPX1* (**C**), *PRDX6* (**D**), or *NRF2* (**E**) in secondary follicles cultured in dECM for 12 days in TCM199.^+^ alone or supplemented with 0.02 μM resveratrol-loaded nanoparticles, 0.2 μM blank nanoparticles or 2 μM nonencapsulated resveratrol. a, b, c Different lowercase letters indicate statistically significant differences between treatments (*P* < 0.05)

After 12 days of culture, follicles cultured with 0.02 μM RLNP showed reduced mRNA expression of *CAT* (*P* < 0.01), *SOD* (*P* < 0.0001), *PRDX6* (*P* < 0.0001), and *NRF2* (*P* < 0.05) compared to the TCM199^+^ control group (*P* < 0.05), although *SOD* levels were similar to those observed in follicles cultured with unencapsulated resveratrol (Fig. 7). No significant differences in *GPX1* expression were observed among treatments (*P* > 0.05).

## Discussion

This study demonstrated that culturing secondary follicles in dECM-based bioscaffolds associated with medium supplemented with RLNP provides a supportive microenvironment for the in vitro maintenance of bovine early follicles. In this study, ovarian dECM was evaluated in relation to a two-dimensional reference condition to examine its ability to support in vitro follicle maintenance. Maintaining ECM integrity during the decellularization process is particularly crucial for functional ovarian tissue engineering, where ECM–follicle interactions play a key role (Wang et al., [9]; Vasse et al., [41]; [42, 43]). In our study, freeze–thaw cycles followed by 9-h incubations in Triton X-100 and SDS effectively removed cells and residual DNA, and yielded scaffolds with preserved macro- and microarchitecture, resembling native tissue. Similar preservation of ovarian microarchitecture has been reported using freeze–thaw cycles combined with Triton X-100 and SDS in ovarian tissue decellularization [36, 44]. Alongside effective cell removal, the preservation of ovarian microstructures and ECM components is a critical determinant of bioscaffold quality following decellularization (Léon-Félix et al., [45]). The GAGs and collagen levels remained comparable to controls and stable during in vitro culture for 10 and 12 days, respectively, despite minor microarchitectural changes. Consistent with this framework, previous ovarian dECM studies report that maintaining collagen- and GAG-rich domains is associated with improved scaffold integrity and cytocompatibility [11]. In our study, follicle viability was maintained throughout the culture period, indicating no apparent cytotoxicity, as similarly reported in ovarian dECM-based follicle culture systems [46].

Due to its preserved porous architecture, decellularized extracellular matrix scaffolds are considered suitable for in vitro culture, as it enables efficient diffusion of culture media and promotes nutrient exchange [46]. Follicles cultured in the 3D system exhibited greater intracellular esterase activity, and reduced membrane damage compared to those maintained in the 2D system under identical conditions. These findings indicate that the 3D system alone already provides improved conditions for follicular maintenance, irrespective of culture medium supplementation. Silva et al. (2024a, b) demonstrated a correlation between non-specific esterase activity and both viability and growth of ovarian follicles in bovine. Accumulating evidence indicates that follicular development relies on dynamic and reciprocal interactions between the follicle and its surrounding]microenvironment (Wang et al., [9]). Thus, dECM scaffolds may offer a supportive mechanical microenvironment by conveying biomechanical cues essential for promoting follicular survival [11]. Furthermore, GAGs and other specific ECM domains have a strong binding affinity for growth factors that can be in vitro released and influence follicular development [18, 47, 48]. The bioactivity of bovine ovarian dECM has already been demonstrated by Laronda et al. [20], who reported estradiol secretion by primary mouse ovarian cells cultured on dECM scaffolds. Similarly, Alaee et al. ([49]) showed that preantral follicles cultured in dECM scaffolds exhibited increased diameter, enhanced antral cavity formation, and higher estradiol and progesterone secretion after 12 days of in vitro culture compared to 2D systems. In addition, porcine ovarian cells seeded onto dECM scaffolds expressed key granulosa cell markers, including STAR, CYP11A1, CYP19A1, AMH, FSHR, and LHR [36], indicating the ability of ECM-based scaffolds ability to drive follicular development in vitro.

In this study, the physicochemical characterization of the nanoparticles demonstrated mean sizes below 150 nm for both formulations, a feature commonly associated with efficient cellular uptake [50]. The PDI values ranged from 0.16 for blank nanoparticles to 0.26 for resveratrol-loaded nanoparticles, indicating moderate size distribution uniformity, which is considered acceptable for drug delivery systems [51]. Both formulations exhibited negative surface charges, with zeta potential values close to − 6 mV. Although low zeta potential magnitudes typically indicate reduced colloidal stability, [64] reported that these formulations maintained colloidal stability for up to 90 days.

Notably, RLNP supplementation of the culture medium in the 3D system enhanced follicular viability across all tested concentrations. The efficacy observed at the lowest tested concentration (0.02 µM) is consistent with previous evidence indicating that nanoencapsulation enables biological responses at low resveratrol exposure levels [52]. Transmission electron microscopy revealed that oocytes from follicles cultured with RLNP had a well-preserved zona pellucida, while granulosa cells showed intact mitochondria and endoplasmic reticulum, with no evident ultrastructural damage. Free resveratrol was included as a reference condition at a concentration commonly used in isolated follicle culture studies [34], whereas RLNP was evaluated across a broader concentration range to explore the effects of nanoencapsulation at lower doses. Several studies have reported that resveratrol enhances ATP production and mitochondrial biogenesis in mammalian granulosa cells, including those from aged cows, thereby improving mitochondrial function and supporting oocyte development in vitro [53, 54]. As a known Sirtuin-1 (SIRT1) activator, resveratrol upregulates SIRT1 expression and increases ATP levels in bovine oocytes, resulting in improved fertilization outcomes [55]. In rats, it also promotes TZP synthesis by increasing cytosolic calcium, activating Calcium/Calmodulin Dependent Protein Kinase II Beta (CaMKIIβ), and releasing actin monomers [56]. In our study, the effects observed with resveratrol encapsulated in polymeric nanoparticles suggest that this drug delivery system may be advantageous, as it can improve the aqueous solubility and bioavailability of resveratrol, enhance its physicochemical stability, and enable targeted and controlled drug release [57, 64].

Resveratrol has been widely reported in the literature as exhibiting a potent antioxidant effect in the ovary [58, 59]. After 12 days of culture, follicles cultured with 0.02 μM RLNP showed reduced mRNA expression of *CAT*, *SOD*, *PRDX6*, and *NRF2* compared to follicles cultured in control medium, although *SOD* levels were similar to those follicles cultured with unencapsulated resveratrol. At first glance, the reduced expression of antioxidant-related genes observed in follicles cultured with RLNP may appear counterintuitive, given the well-established antioxidant properties of resveratrol. Antioxidant gene transcription, particularly through NRF2-dependent pathways, is tightly regulated by intracellular ROS levels, and a decrease in transcription may reflect a reduced requirement for endogenous antioxidant defenses in a less oxidative microenvironment [60, 61]. This interpretation is biologically plausible, as resveratrol is known to act as a free radical scavenger and modulate redox signaling pathways. As a direct antioxidant agent, resveratrol has well-documented antioxidant activity and has been shown to reduce intracellular ROS levels and oxidative damage in granulosa cells, including in bovine models [58, 62, 63].

In conclusion, the decellularized bovine ovarian tissue exhibited minimal residual cellular content and well-preserved ECM ultrastructure. The 3D culture system supported follicular viability and ultrastructural preservation, especially when supplemented with RLNP. At 0.02 µM, RLNP preserved follicular ultrastructure, maintained essential features such as the zona pellucida, granulosa cells, and intracellular organelles. Moreover, supplementation with 0.02 µM RLNP was associated with lower mRNA expression of the antioxidant-related genes CAT, SOD, GPX1, PRDX6, and NRF2. Future studies incorporating canonical follicular gene expression and hormone secretion analyses will be required to assess follicular cell phenotypes and endocrine function.

## Data Availability

All data produced or analyzed during this study are included in this article and can be shared upon reasonable request to the corresponding author.
